# NOX1 Supports the Metabolic Remodeling of HepG2 Cells

**DOI:** 10.1371/journal.pone.0122002

**Published:** 2015-03-25

**Authors:** Katharina Bertram, Cristina-Maria Valcu, Michael Weitnauer, Uwe Linne, Agnes Görlach

**Affiliations:** 1 Experimental and Molecular Paediatric Cardiology, German Heart Centre Munich at the Technical University Munich, Lazarettstr. 36, Munich, Germany; 2 Chemistry Department—Mass Spectrometry, Philipps-University Marburg, Hans-Meerwein-Strasse, Marburg, Germany; University of Iowa, UNITED STATES

## Abstract

NADPH oxidases are important sources of reactive oxygen species (ROS) which act as signaling molecules in the regulation of protein expression, cell proliferation, differentiation, migration and cell death. The NOX1 subunit is over-expressed in several cancers and NOX1 derived ROS have been repeatedly linked with tumorigenesis and tumor progression although underlying pathways are ill defined. We engineered NOX1-depleted HepG2 hepatoblastoma cells and employed differential display 2DE experiments in order to investigate changes in NOX1-dependent protein expression profiles. A total of 17 protein functions were identified to be dysregulated in NOX1-depleted cells. The proteomic results support a connection between NOX1 and the Warburg effect and a role for NOX in the regulation of glucose and glutamine metabolism as well as of lipid, protein and nucleotide synthesis in hepatic tumor cells. Metabolic remodeling is a common feature of tumor cells and understanding the underlying mechanisms is essential for the development of new cancer treatments. Our results reveal a manifold involvement of NOX1 in the metabolic remodeling of hepatoblastoma cells towards a sustained production of building blocks required to maintain a high proliferative rate, thus rendering NOX1 a potential target for cancer therapy.

## Introduction

Reactive oxygen species (ROS) act as signaling molecules in the regulation of various physiological and pathological processes in almost all tissues [1]. NADPH oxidases are important sources of ROS which are involved as second messengers in the regulation of gene expression as well as in cell proliferation, differentiation, migration and death. To date, 7 homologous NADPH oxidase enzymes have been identified which mainly differ in the expression of the catalytic NOX subunits, termed NOX1 to NOX5, and DUOX1/2. NOX2 is identical to the previously characterized gp91phox protein of the leukocyte NADPH oxidase [2].

Among other pathologies, malignant transformation and tumor progression have been associated with dysregulated ROS production and members of the NOX family have been previously linked with different types of cancer [3,4]. In particular, NOX1 has been studied in relation with oncogenic Ras transformation [5,6] and was shown to be involved in the regulation of cell proliferation and migration (reviewed by [3,4]).

The NOX1 catalytic subunit of NADPH oxidase associates with the stabilizing subunit p22phox, the activator subunit NOXA1 and the organizing subunit NOXO1, and requires Rac1 for activation [7], but can also interact with p47phox and p67phox characteristically involved in the NOX2-dependent NADPH oxidase [8]. The enzyme is involved in the signaling cascades of several stimuli such as tumor necrosis factor (TNFα), platelet-derived growth factor (PDGF), epidermal growth factor (EGF), basic fibroblast growth factor (bFGF) and angiotensin-II (reviewed in [8]).

NOX1 has been reported to be over-expressed in colon [9], gastric [10], prostate [11], bladder [12], kidney [13], breast and ovarian cancer [14]. A correlation between NOX1 levels and the tumor grade/stage was observed in bladder cancer, though not in colon cancer [15]. In Ras-transformed cells, NOX1-induced Rho inactivation causes the disruption of actin stress fibers and focal adhesions [16]. The mechanism underlying the Ras-dependent transcriptional activation of NOX1 involves the MEK-ERK-dependent phosphorylation of GATA-6 [17]. On the other hand, NOX1 has been reported to impair acetylation of the tumor suppressor p53 and its pro-apoptotic transcriptional activity through a mechanism involving SIRT1 deacetylase, thus inhibiting apoptosis [18].

In human hepatoblastoma cells HepG2, NOX1 knockdown prevents autocrine growth through decreasing EGFR and TGF-α in a p38 MAPK and AKT dependent manner [19]. In order to gain insight into the role of NOX1 in hepatic tumors, we investigated the proteome of HepG2 cells stably expressing shRNA against NOX1. We identified several protein functions dysregulated in the presence of reduced NOX1 levels, providing interesting indications regarding the involvement of NOX1 in the regulation of tumor cell metabolism.

## Material and Methods

### Biological material

Hepatoblastoma (HB) cells HepG2 (ATCC HB-8065) were maintained in DMEM medium (PAA, Coelbe, Germany) containing 4.5 g/l glucose, supplemented with 10% FCS (PAN Biotech, Aidenbach, Germany), 100 U/ml penicillin (PAA) and 100 μg/ml streptomycin (PAA) in a humidified incubator at 37°C and 5% CO_2_. Human hepatoma cells HuH-7 [20] were cultured in DMEM medium (Biochrom, Berlin, Germany) containing 4.5 g/l glucose and 580 mg/l stable L-glutamine, supplemented with 10% FCS (PAN), 110 mg/l sodium pyruvate, 100 U/ml penicillin and 100 μg/ml streptomycin (all PAA) in a humidified incubator at 37°C and 5% CO2.

In order to generate HepG2 cell lines with differential levels of NOX1, psiStrike (Promega, Mannheim, Germany) plasmids encoding a specific short hairpin RNA against NOX1 (shNOX1) or a random control sequence (shCtr) [21] were used (shNOX1_I:ACCGCACCGGTCATTCTTTATATTTGTGTAGTATAAAGAATGACCGGTGCTTTTTC; shNOX1_II: ACCGTTGGTCATGCAGCATTAATTTGTGTAGTTAATGCTGCATGACCAACTTTTTC, shCtr: ACCGTCTCCGAACGTGTCACGTTTCAAGAGAACGTGACACGTTCGGAGAATTTTTC).

For generation of stable clones, HepG2 cells were transfected with shCtr or shNOX1_I using Fugene HD transfection reagent (Roche, Penzberg, Germany). After 48 h cell culture media was changed and geneticin (G418, 100 μg/ml, Life Technologies, Darmstadt, Germany) was added. After two weeks of selection in the presence of geneticin, single cell clones were established by plating 10 μl cell suspension onto a 96 well plate at a concentration of 2 cells/ml. Cell cultures derived from more than one colony were discarded. Cells were further cultivated in standard DMEM medium supplemented with geneticin.

### Sample preparation

Stable cell lines were grown to 80% confluence in 150 mm Petri dishes. Sample preparation and protein extraction were optimized using non-transfected HepG2 cells. The optimizations regarded cell harvesting (scraping vs. *in situ* lysis), cell disruption (freeze/thaw vs. tip sonication), and washing and lysis buffer. All chemicals were purchased from GE Healthcare (Munich, Germany) unless otherwise specified. Sample buffer components tested included the reducing/oxidizing agent (DTT vs. HED), spermine (Sigma-Aldrich, Taufkirchen, Germany), benzonase (Sigma-Aldrich), as well as the detergent assortment of the ProteoPrep detergent sampler kit (Sigma-Aldrich). Following the optimized protocols, cells were washed three times with Tris buffered sucrose solution containing protease and phosphatase inhibitors (2 mM PMSF—Sigma-Aldrich, 1 mM NaF—Sigma-Aldrich and 0.2 mM Na_3_VO_4_—Merck, Darmstadt, Germany) and detached with a cell-scraper in the same buffer. Cells were pelleted, then resuspended in 1 ml lysis buffer (7 M urea, 2 M thiourea, 4% CHAPS, 100 mM DTT, 25 mM spermine and 0.5% Pharmalyte 3–10) and disrupted by tip sonication on ice (5 x 30 second cycles 90% of time at 70% power). After 1 h incubation on ice, the protein extract was centrifuged for 30 min at 40000 *x g* and 4°C and proteins were quantified using RC-DC Quant kit (Bio-Rad, Munich, Germany).

### Two-dimensional electrophoresis

Cells were allowed to accumulate mutations during at least 8 independent passages in order to obtain samples approaching biological replicates. A pilot experiment was performed with 6 samples per gradient and power analysis was employed to calculate the optimal sample size. It was estimated that 7 samples were necessary in order to reach an experimental power of 0.8 to detect fold changes of 2 for 96% of the spots and fold changes of 1.5 for 72% of the spots at a p-value threshold of 0.05. Thus, subsequent differential display 2DE experiments employed 7 biological replicates per cell line.

Samples were cup loaded (500 μg protein/strip) on 4–7 (24 cm) and 6–11 (18 cm) IPGs (GE Healthcare) rehydrated over night with rehydration buffer (7 M urea, 2 M thiourea, 2% CHAPS, 100 mM HED, 0.5% Pharmalyte 3–10, 0.002% bromophenolblue and 10% isopropanol for basic IPGs) and focused on an Ettan IPHphor 3 Cup Loading Manifold (GE Healthcare) for 64 kVhr and 50 kVhr, respectively. Strips were equilibrated prior to the second dimension two times for 15 min under gentle shaking at room temperature in equilibration solution (50 mM Tris-HCl pH 8.8, 6 M urea, 10% SDS, 30% w/v glycerol, 0.002% bromophenolblue) containing 2% DTT and 4% iodoacetamide, respectively. Equilibrated strips were sealed on top of 10% polyacrylamide gels with 0.5% low melting agarose solution. The second dimension was run over night in an Ettan Dalt6 chamber (GE Healthcare). 2DE gels were stained with Ruthenium (II) tris (bathophenantroline disulfonate) fluorescent stain (RubiLAB, Burgdorf, Switzerland) and documented with a Typhoon Trio+ scanner (GE Healthcare) at 100 μm resolution under 610 nM emission, 532 nm laser, 600 V PTM.

### Image and 2DE data analysis

2DE image analysis was performed with Progenesis SameSpots (Nonlinear Dynamics, Newcastle upon Tyne, UK). Relevant spot parameters were exported and stored in an MS Access database. Statistical analysis was performed on normalized spot volumes with R 2.14.0. [22] using the following packages: RODBC [23], nlme [24], effects [25], lattice [26], Hmisc [27], multcomp [28], ssize [29]. Normalization was visually checked by inspection of box-plot distribution of normalized spot volumes among samples. Normalized spot volume distributions did not differ between runs or edited vs. non-edited spots.

Differential expression was established based on statistically significant differences between shNOX1 and shCtr cells (Welsch t-test p-values adjusted for false discovery rate, FDR) as well as fold regulations exceeding the intrinsic spot characteristic biological variability [30], as described below.

The level of intrinsic spot volume variation was assessed by non-parametric bootstrap (sampling with repetition of 2 random samples of 6 to 7 biological replicate control samples, 1000 times per spot) and expressed as the 95% quantile of the distribution of fold differences between the two random samples. This represents the upper level of expected variance which naturally appears in control samples for each spot. The intrinsic spot volume variance expressed as the 95% quantile of the fold changes distribution was modeled using a glm with log transformed average spot volume and spot position on the 2DE gel as predictors. The intrinsic levels of variation in protein abundance thus predicted were used as thresholds for the selection of biologically relevant changes in protein abundance between control and NOX1 reduced samples. Significant statistical differences in protein abundance were assessed using Welsch t-tests on normalized spot volumes and reported as FDR adjusted p-values. Protein spots which simultaneously fulfilled both criteria were selected as differentially expressed and were further validated against non-transfected HepG2 control cells as well as against an independent shNOX1 line.

### Mass spectrometry

Protein spots were excised from preparative 2DE gels loaded with 800 μg protein and stained with Colloidal Coomassie G-250 staining according to Anderson [31]. The gel plugs were prepared prior to MS analysis as previously described [30].

The mass spectrometric analysis of the samples was performed using a LTQ-FT Ultra mass spectrometer (ThermoScientific, Dreieich, Germany). A nanoHPLC system consisting of ULTIMATE, SWITCHOS and FAMOS (Dionex, Idstein, Germany), equipped with a homemade nano 5 μm C18 RP column (10 cm in length, 75 μm diameter) was connected online to the mass spectrometer through a nanospray ion source. 15 μl of the tryptic digest (25 μl each) were usually injected onto a C18 pre-concentration column. Automated trapping and desalting of the sample was performed at a flow rate of 30 μl/min using water/0.05% formic acid as solvent.

Separation of the tryptic peptides was achieved with the following gradient of water/0.045% formic acid (solvent A) and 80% acetonitrile/0.05% formic acid (solvent B) at a flow rate of 300 nL/min: holding 4% B for five minutes, followed by a linear gradient to 45% B within 30 minutes and linear increase to 95% solvent B in additional 5 minutes. The column was connected to a stainless steel nanoemitter (Proxeon, Denmark)) and the eluent sprayed directly towards the heated capillary of the mass spectrometer using a potential of 2300 V. A survey scan with a resolution of 100,000 within the FT-ICR mass analyzer was combined with three data-dependent MS/MS scans with dynamic exclusion for 30 s using CID within the linear ion-trap.

Thermo´s. raw files were sent to Mascot using LCQ-DTA software provided by Thermo. MS spectra were searched against nrNCBI database (15 033 251 sequences; November 2011) using MASCOT 2.2 (Matrix Science Ltd., London, UK). Mass tolerance for precursor ions was set to 5ppm and for fragment ions to 0.6 Da; up to one missed cleavages were allowed; fixed modification was carbamidomethylation of cysteine; the option “error tolerant search” was enabled. MASCOT hits were considered confident when based on at least three MS/MS spectra with peptide ions scores above 20 or two spectra with scores higher than 40.

### Western Blot

Up to 50 μg of protein lysates were separated by SDS-PAGE on 10% polyacrylamide-gels as previously described [32]. Primary antibodies were applied at a dilution of 1:1000 overnight at 4°C. Primary antibodies were acquired as follows: GDH1 (Abgent, Heidelberg, Germany), UDPGP (Abnova, Heidelberg, Germany), Fascin (Abcam, Cambridge, UK), I2PP2A (SET protein, Santa Cruz Biotechnology, Heidelberg, Germany), HSPc71 (Cell Signaling, Danvers, MA, United States), AFP (Proteintech Group, Manchester, United Kingdom), Acsl1 and HMGCS1 (Sigma Aldrich, Munich, Germany). Primary antibody against NOX1 was raised against the NOX1 peptide H2N-CAESFEMWDDRDSH-CONH2, (Eurogentec, Cologne, Germany).

### ROS production measurements

ROS production was determined using the cell permeant fluoroprobe 5-(and-6)-chloromethyl-2’,7’-dichlorodihydrofluorescein diacetate acetyl ester (CM-H2DCFDA, Invitrogen, Darmstadt, Germany) as described before [32].

### Glycogen assay

HepG2 cells were transiently with shRNA plasmids transfected using Fugene HD. Transfection was carried out according to manufacturer’s instructions using 3 μg DNA and 9 μl Fugene per 6 cm dish. At 48 h after transfection cells were harvested. Each sample was performed in duplicate. One replicate was used for protein isolation, and the second for glycogen assay. Protein concentration was determined using Roti-Quant (Carl Roth, Karlsruhe, Germany). For glycogen measurements, cells were homogenized in Milli-Q water and boiled for 5 min. After a 10 min centrifugation at 13,000 *x g*, glycogen levels were determined in the supernatant using Glycogen Assay Kit (Sigma-Aldrich, Munich, Germany) according to manufacturer’s instructions.

Data were analyzed using a linear mixed effect model [33] with the relative glycogen abundance as dependent variable, clone (non-transfected HepG2 control vs. shCtr vs. shNOX1) as predictor and replicate (N = 4) as random factor. In order to further test for group differences we applied post-hoc comparisons between shCtr vs. non-transfected HepG2 control and shCtr and shNOX1. To assess the correlation between glycogen and NOX1 levels we additionally used shNOX_II and applied a linear mixed effect model [33] with Gaussian error distribution and replicate as random factor (N = 4).

### Measurement of metabolic activity

Metabolic activity of stable HepG2 clones was determined using AlamarBlue (Invitrogen). HepG2 cells were seeded at a density of 250 cells/well in 96 well plates in the presence of AlamarBlue. AlamarBlue fluorescence was measured over a time period of one week in daily basis in a microplate reader (Tecan, Crailsheim, Germany) at 555nm excitation and 585 nm emission wavelength. AlamarBlue fluorescence increases lineary with the availability of reduction equivalents produced by the metabolism of cells therefore the slope is an indicator for the metabolic rate [34]. The difference in slopes between NOX1 depleted cells and control cells was tested using a mixed effect model with replicate (N = 3) as random factor.

## Results

### Protein expression changes in NOX1 reduced cells

**Fig 1:**
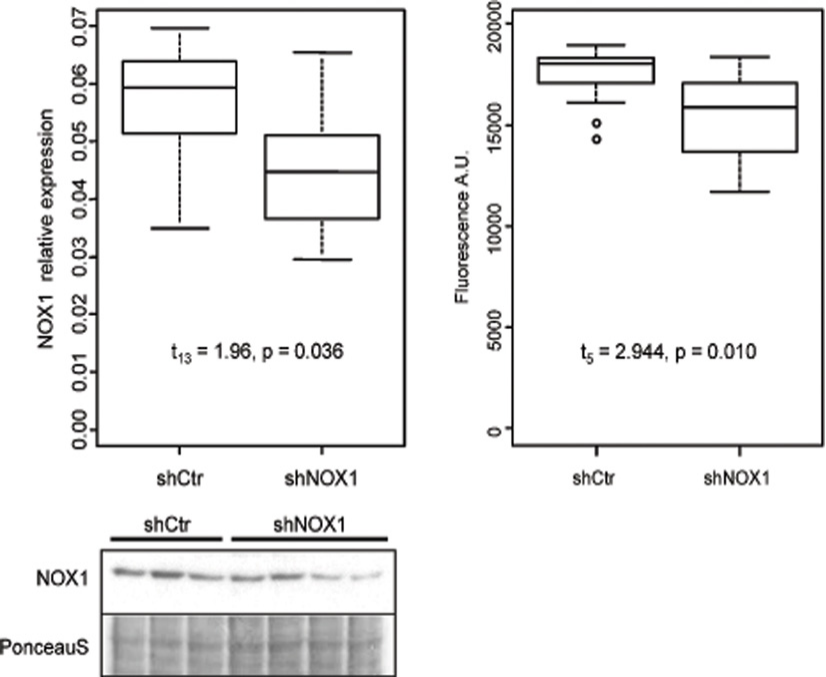
NOX1 and ROS levels are decreased in HepG2 cells expressing shNOX1. NOX1 levels were determined by Western blot analysis in HepG2 cells stably expressing shRNA against NOX1 or a control shRNA. The image exemplifies a representative NOX1 Western blot. Relative protein expression is expressed as ratio between the Western blot intensity and total protein content of the sample determined from the PonceauS staining (left). Differences between groups were assessed using Welsch t-test (N_shCtr_ = 8, N_shNOX1_ = 7). The dependence of the abundance of the dysregulated proteins upon NOX1 protein levels was tested in a mixed effect model with spot ID as random factor. ROS production was measured using CM-H2DCFDA assay (right). Differences in ROS levels between NOX1 reduced HepG2 cells and control cells were tested in a mixed effect model with cell type nested in replicates (N = 3).

In order to gain insight into the involvement of NOX1 in controlling cellular processes in human hepatoblastoma cells, we used HepG2 cells stably expressing shRNA against NOX1 or a control shRNA. Compared to cells expressing shCtr, cells expressing shNOX1 showed a 20% reduction of NOX1 protein levels as determined by Western blot analysis (t_13_ = 1.96, p = 0.036, Fig. 1).

Differences in ROS levels between NOX1 reduced HepG2 cells and control cells were measured as described in above and tested using a mixed effect model with cell type nested in replicates (N = 3). Based on this model, we estimated a mean reduction in ROS levels by 13% in NOX1 reduced cells compared to control cells (t_5_ = -2.944, p = 0.010) indicating that the reduction of NOX1 was functionally relevant (Fig. 1, right side). Levels of other NOX proteins such as NOX2, NOX4 or NOX5, could not be detected or did not differ between control and NOX1 reduced cells (data not shown).

**Fig 2:**
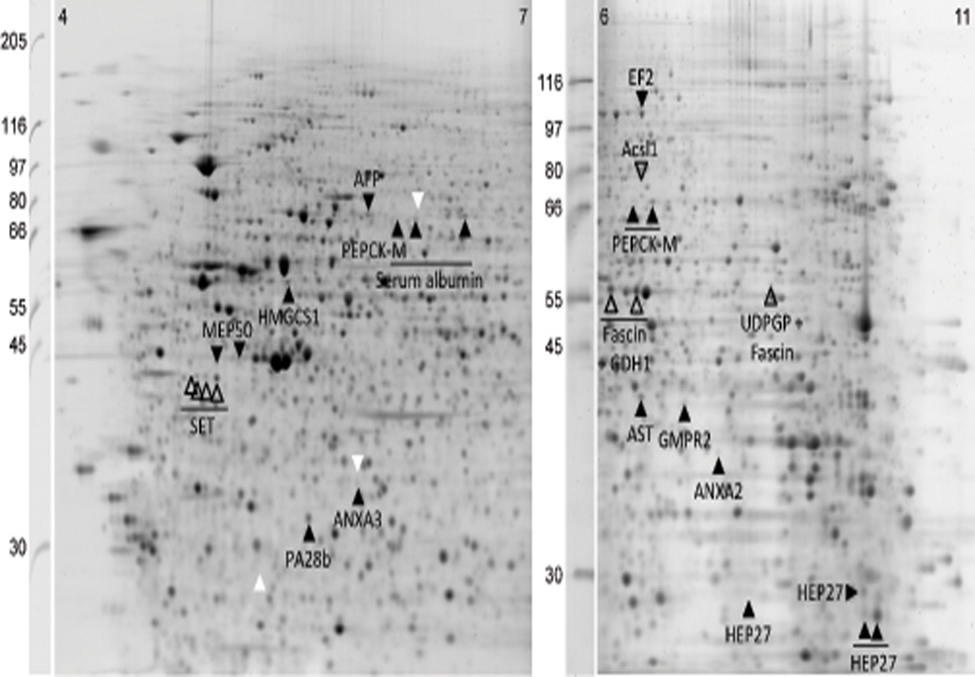
2DE map displaying proteins differentially expressed in control vs. NOX1 reduced HepG2 cells. Empty arrows denote up-regulated protein functions, filled arrows denote down-regulated protein functions. White arrows denote differentially regulated but un-identified protein spots.

**Fig 3:**
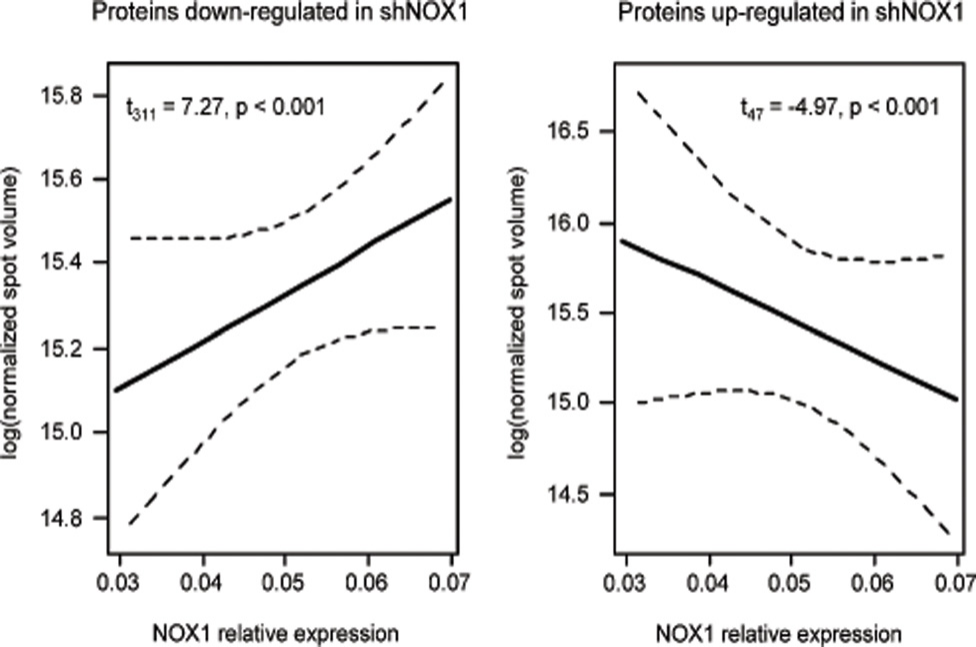
Abundance of differentially expressed proteins depends upon NOX1 relative expression (plotted are 95% CI).

Following 2DE separation of proteins isolated from these cell lines, a total of 2222 and 1601 protein spots were identified on the 4–7 and 6–11 IPGs, respectively. Of those, 29 and 16, respectively, had significantly different expression levels in NOX1 reduced cells compared to control cells at a fold difference exceeding the intrinsic level of biological variation as described in above. After validation against the non-transfected (wild type) HepG2 cells, 16 spots from the acidic gradient and 14 from the basic gradient were selected as dysregulated in NOX1 reduced cells (Fig. 2). Of them, 26 were more abundant in control cells while 4 were more abundant in NOX1 reduced cells. The dependence of the abundance of these proteins upon NOX1 protein levels was found highly significant for both up-regulated and down-regulated proteins (t_47_ = -4.97, p<0.001 and t_311_ = 7.27, p<0.001 respectively) (Fig. 3).

### Identification of differentially expressed proteins

**Fig 4:**
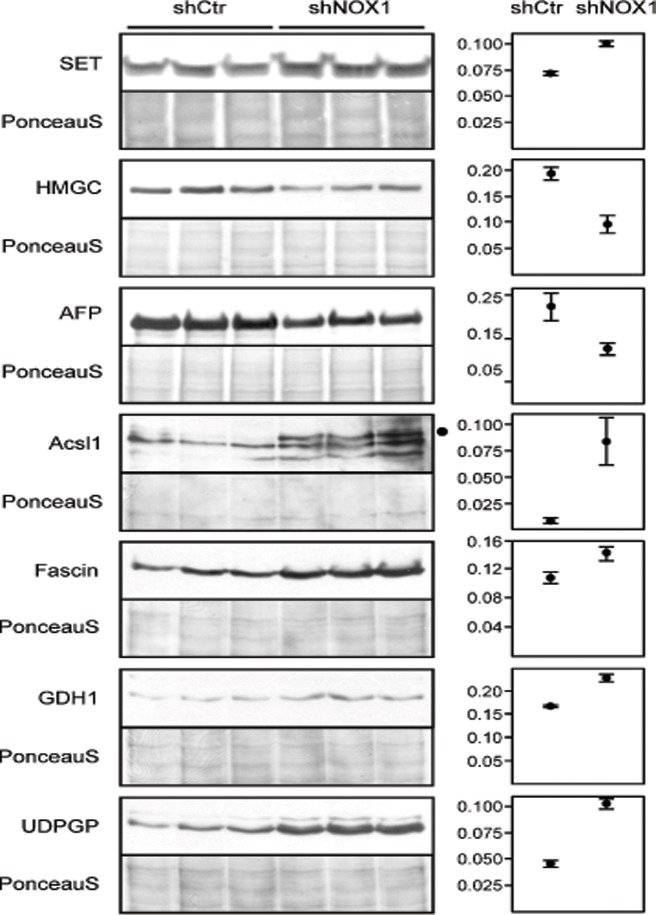
Western blot validation of NOX1 regulated proteins. The major components of the 2DE spots which contained complex proteins mixtures were subjected to validation through Western blot analysis. Seven of the proteins were confirmed to be differentially expressed in control vs. NOX1 reduced HepG2 cells. Plotted are the mean and SEM of WB intensities normalized to the total protein content of the sample determined from the PonceauS staining. SET—protein SET, HMGC—HMG-CoA-synthase, AFP—Alpha-fetoprotein, Acsl1—Long chain fatty acid-CoA ligase, GDH1—Glutamate dehydrogenase 1, UDPGP—UTP-glucose-1-phosphate uridylyltransferase. Statistical results are presented in Table 1.

Protein spots identified as differentially expressed between NOX1 reduced cells and control cells were excised from both gels and analyzed by tandem mass spectrometry as described above. Valid identifications were obtained for 27 of the 30 analyzed spots (Table 1 and S1 Table). Eighteen spots were mixtures of two to eight proteins and were further investigated as described previously [35]. A comparison of spectral counts and spectral abundance factors [36] of the hit lists obtained for each spot from NOX1 reduced and control samples enabled the identification of regulated proteins in 14 of these spots (S1 Table). Further validation by Western blot was performed as described in above for the major hits in the four remaining spots. The major protein components of the spots for which the MS analysis was successful in only one of the samples and of the spots for which protein regulation according to spectral counts and spectral abundance factors were in disagreement with the spot regulation apparent from the 2DE gel were also validated by Western blot analysis. Of the eight protein functions verified by Western blot, seven were validated (Fig. 4) and one (heat shock protein cognate 71) was invalidated.

**Table 1:** Protein functions dysregulated in NOX1 reduced cells.

Spot	Best homologue		Gene	Accession number	shNOX1 regulation (2DE)		Total score	Matched spectra	Unique peptindes	Sequence coverage	WB validation	
	Protein name	Abbrev.			Fold	p value^a^					Fold	p value^b^
1	Protein SET	SET	SET	Q01105	0.5	0.008	138	11	5	20%	1.4	0.001
2	Protein SET	SET	SET	Q01105	0.4	0.001	91	10	5	22%		
3	Protein SET	SET	SET	Q01105	0.3	0.003	48	4	3	14%		
4	Protein SET	SET	SET	Q01105	0.3	0.007	53	5	3	14%		
5	Methylosome protein 50	MEP50	WDR77	Q9BQA1	0.5	0.048	184	17	5	19%		
6	Methylosome protein 50	MEP50	WDR77	Q9BQA1	0.6	0.002	723	59	11	44%		
8	Proteasome activator PA28 subunit beta	PA28b	PSME2	Q9UL46	0.5	0.001	453	52	12	41%		
9	Annexin A3	Annexin A3	ANXA3	P12429	0.5	0.023	919	100	16	50%		
11	Hydroxymethylglutaryl-CoA synthase, cytoplasmic	HMG-CoA synthase	HMGCS1	Q01581	0.6	0.023	843	63	21	49%	0.5	0.007
12	Alpha-fetoprotein precursor	Alpha-fetoprotein	AFP	P02771	0.6	0.044	3672	395	40	75%	0.6	0.037
13	Phosphoenolpyruvate carboxykinase [GTP], mitochondrial	PEPCK-M	PCK2	Q16822	0.5	0.007	384	28	9	16%		
	Serum albumin	Serum albumin	ALB	P02768			61	6	5	8%		
14	Serum Albumin	Serum albumin	ALB	P02768	0.5	0.000	967	152	32	54%		
16	Serum Albumin	Serum albumin	ALB	P02768	0.5	0.001	2741	425	45	71%		
17	Elongation factor 2	EF2	EEF2	P13639	0.6	0.007	675	67	30	36%		
18	Long chain fatty acid-CoA ligase 1 (Acsl1)	Acsl1	ACSL1	P33121	2.6	0.002	587	46	14	26%	9.8	0.037
19	Phosphoenolpyruvate carboxykinase [GTP], mitochondrial	PEPCK-M	PCK2	Q16822	0.4	0.024	142	4	3	5%		
20	Phosphoenolpyruvate carboxykinase [GTP], mitochondrial	PEPCK-M	PCK2	Q16822	0.5	0.002	1201	61	15	24%		
21	Fascin	Fascin, p55	FSCN1	Q16658	2.3	0.001	11324	770	31	62%	1.3	0.027
	Glutamate dehydrogenase 1, mitochondrial	GDH1	GLUD1	P00367			417	95	21	41%	1.4	0.006
22	Fascin	Fascin, p55	FSCN1	Q16658	2.1	0.001	9861	593	37	74%		
	Glutamate dehydrogenase 1, mitochondrial	GDH1	GLUD1	P00367			180	30	15	29%		
23	UTP—glucose-1-phosphate uridylyltransferase	UDPGP	UGP2	Q16851	1.7	0.007	2302	161	18	38%	2.2	0.000
	Fascin	Fascin, p55	FSCN1	Q16658			244	38	7	15%		
24	Aspartate aminotransferase, cytoplasmatic	cAspAT	GOT1	P17174	0.5	0.001	10141	736	41	77%		
25	Guanosine monophosphate reductase 2	GMP reductase	GMPR2	Q6PKC0	0.6	0.027	750	86	19	53%		
26	Annexin A2 isoform 2	Annexin A2	ANXA2	P07355	0.3	0.002	2994	227	31	77%		
27	Dicarbonyl reductase HEP27	HEP27	DHRS2	Q13268	0.5	0.002	530	39	20	68%		
28	Dicarbonyl reductase HEP27	HEP27	DHRS2	Q13268	0.5	0.004	8124	441	32	85%		
29	Dicarbonyl reductase HEP27	HEP27	DHRS2	Q13268	0.4	0.001	5456	304	22	69%		
30	Dicarbonyl reductase HEP27	HEP27	DHRS2	Q13268	0.4	0.000	10917	711	47	87%		

^a^ adjusted for FDR,

^b^ one tailed Welch t test for unequal variance.

Spots 1–4 represent most likely isoforms of the same protein as suggested by their appearance on the 2DE gel (Fig. 2). Although no identification was obtained for the spots excised from control gels, on which all four spots had higher volumes, the same protein (SET) was identified in all spots excised from NOX1 reduced gels, suggesting an up-regulation of the protein in these cells. Western blot analysis confirmed that SET protein is up regulated in NOX1 reduced samples (Fig. 4), indicating that the major component of spots 1–4 responsible for the apparent down regulation of this protein spot has not been identified.

Altogether 17 protein functions were identified as differentially expressed between NOX1 reduced and control cells.

### Profiling of regulated proteins

The 17 differentially expressed proteins were functionally classified into proteins involved in cellular metabolism (7 functions), protein synthesis and turnover (3 functions), intra- and extracellular transport (4 functions), cytoskeleton organization (1 function), DNA replication and repair (1 function) and detoxification (1 function) (S1 Fig.). Protein localization was distributed as follows: 2 extracellular, 3 associated to the plasma membrane, 6 cytoplasmic, 4 mitochondrial and 2 intra nuclear (S2 Fig.).

### Glycogen levels and metabolic activity in NOX1 reduced cells

One of the enzymes which increased in abundance in NOX1 reduced cells is UTP-glucose-1-phosphate uridylyltransferase (UDPGP, EC = 2.7.7.9), suggesting an involvement of NOX1 in modulating glycogen biosynthesis in HepG2 cells. This protein was also increased in HuH-7 liver tumor cells transiently transfected with two different shRNAs against NOX1 compared to control cells (S3 Fig.).

**Fig 5:**
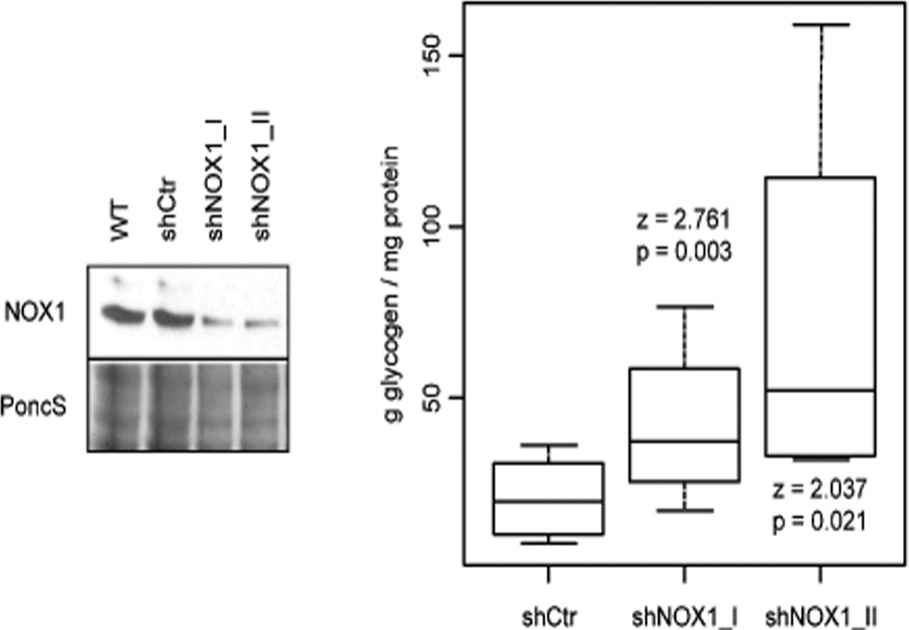
NOX1 regulates glycogen content in HepG2 cells. Glycogen levels were assessed in HepG2 transiently transfected with two shRNAs against NOX1 or with control shRNA. NOX1 reduced HepG2 cells produce more glycogen than shCtr expressing cells (right). Differences in glycogen levels were tested in a linear mixed effect model with cell line as predictor and replicate (N = 4) as random factor (one-tailed post-hoc z test). NOX1 depletion was confirmed be Western blot analysis (left).

In order to verify that the differential regulation of UDPGP actually translated into differences in the levels of glycogen, we measured the glycogen content of HepG2 cells transiently transfected with two different shRNAs against NOX1 or with control shRNA. The glycogen content of NOX1 reduced cells was elevated 2.7 (z = 2.037, p = 0.021) and 2 fold (z = 2.761, p = 0.003), respectively as compared to control cells (Fig. 5). In addition, overall metabolic activity was determined using the Alamar Blue assay [34]. Compared to control cells, metabolic activity was decreased in NOX1 reduced HepG2 cells. (S4 Fig.).

## Discussion

The NADPH oxidase subunit NOX1 has been related to tumor biology in several cancer entities [3,5,6]. However, the pathways which are controlled by NOX1 in tumor cells are not well defined. In this study we aimed to identify cellular processes dependent on the presence of NOX1 in HepG2 cells. Using differential display 2DE followed by MS/MS analysis we identified 17 protein functions which were dependent on the level of NOX1 expression. Several of them were involved in glucose, glutamine, lipid and nucleotide metabolism, providing support for a possible role of NOX1 in metabolic remodeling of hepatic tumor cells.

### NOX1 and tumor specific metabolic remodeling

Tumor cells are known to exhibit a metabolic shift towards increased glycolysis with most of the resulting pyruvate being converted to lactate, even under aerobic conditions, while the tricarboxylic acid (TCA) cycle and the oxidative phosphorylation activity are reduced. This phenotype is known as the Warburg effect [37]. Tumor cell metabolic remodeling also includes increased lipid synthesis and decoupling of glucose and glutamine utilization yielding to a sustained production of amino acids and nucleotides, aimed to support an increased proliferation rate [38]. In this study, 2DE-based proteomic profiling of HepG2 cells indicated that NOX1 controls several proteins relevant for regulating glucose and glutamine metabolism as well as lipid and nucleotide synthesis, providing support for a regulatory function of NOX1 in hepatic tumor metabolism.

### Glucose metabolism

**Fig 6:**
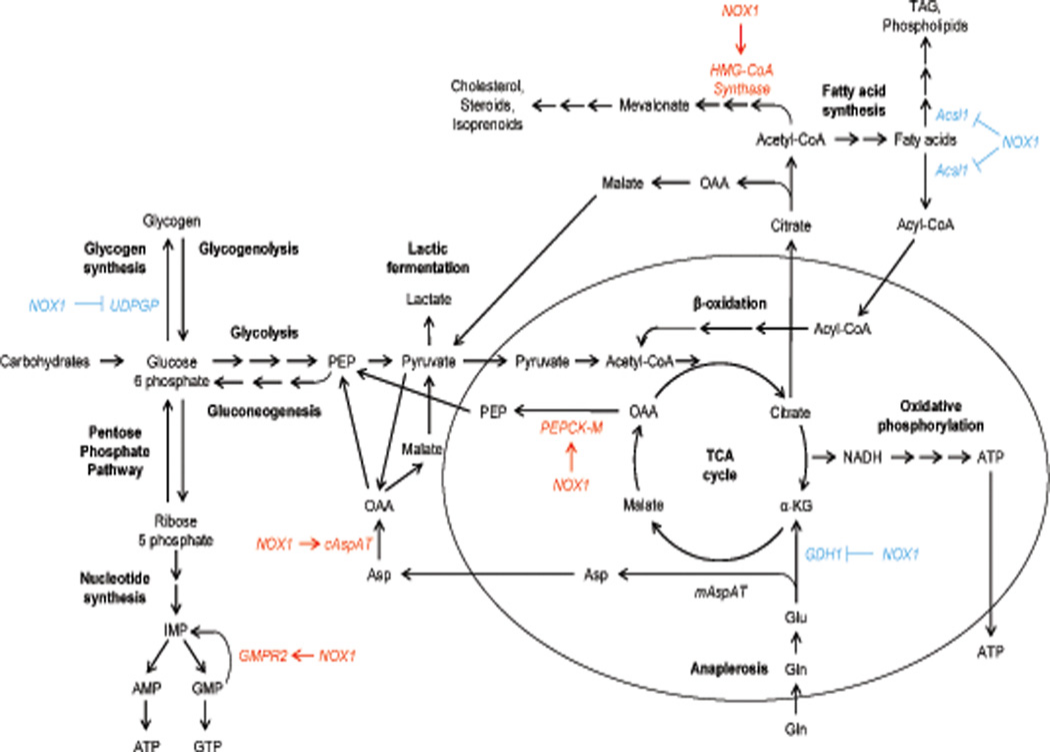
Overview of the metabolic pathways regulated by NOX1. Proteins differentially expressed in control vs. NOX1 reduced HepG2 cells are involved in glucose, glutamine, nucleotide and lipid metabolism. α-KG—α-ketoglutarate, OAA—oxaloacetic acid, PEP—phosphoenolpyruvate, TAG—triacylglycerol, TCA cycle—tricarboxylic acid cycle.

To increase the availability of glucose for glycolysis, tumor cells typically inhibit glucose storage into glycogen. Our results showed that a decrease in NOX1 expression induces in both HepG2 and HuH-7 cells an increase in the abundance of UTP-glucose-1-phosphate uridylyltransferase (UDPGP, EC = 2.7.7.9) (Fig. 4 and S3 Fig.), which catalyzes the synthesis of UDP glucose, a glucosyl donor and direct precursor for glycogen biosynthesis (Fig. 6). UDPGP has previously been found down regulated in hepatocellular carcinoma (HCC) as compared to normal liver tissue [39], while low glycogen content has been associated in hepatoblastoma (HB) with rapid tumor growth [40] and poor outcome [41]. Indeed, reduction of NOX1 enhanced glycogen levels in shNOX1 cells (Fig. 5). In support, NOX3, another NOX homologue, has previously been shown to mediate a decrease in glycogen levels in response to TNF-α in HepG2 cells [42] and NOX suppression was shown to decrease glucose uptake and lactate generation in pancreatic tumor cells [43]. Collectively these data indicate that NOX1 has a role in limiting glycogen synthesis in hepatic tumors implicitly increasing glucose availability for glycolysis.

Reduction of NOX1 also decreased the expression of mitochondrial phosphoenolpyruvate carboxykinase (PEPCK-M, EC = 4.1.1.32) which converts oxaloacetic acid (OAA) to phosphoenolpyruvate (PEP) thus re-channeling the mitochondrial intermediates of the TCA cycle into the cytosolic pool of glycolytic intermediates. Although PEPCK-M has been found down regulated in HCC [44], most tumor cell lines express the enzyme [45] and increased PEPCK-M activity has been observed in lung cancer cells which were proposed to use this reaction to replenish the cellular PEP pool from the lactate accumulated as a result of glycolysis [46]. PEP can then be directed for example towards the pentose phosphate pathway, for NADPH generation and nucleotide synthesis (Fig. 6). Recently, PEPCK-M up-regulation has been shown to promote metabolic adaptation to nutrient availability in cancer cells as a novel pro-survival mechanism [45] and our results suggest an involvement of NOX1 in mediating this regulation.

### Glutamine metabolism

In addition to glucose metabolism, our results suggest an involvement of NOX1 in regulating glutamine metabolism in HepG2 cells. We found that NOX1 levels were inversely correlated to the levels of mitochondrial glutamate dehydrogenase (GDH1, EC = 1.4.1.3) in HepG2 and HuH-7 cells (S3 Fig.). This may result in limited oxidative phosphorylation since GDH1 is responsible for the oxidative deamination of glutamate allowing for the use of glutamate and glutamine as substrates for ATP production in oxidative phosphorylation (Fig. 6). In line, previous reports showed that glutamate metabolism is often dysregulated in hepatoma [47], and the levels of mitochondrial GDH1 are decreased as compared to normal liver and inversely correlated with tumor aggressiveness [47,48].

In both normal and tumor cells, glutaminolysis provides nitrogen for the synthesis of purines, pyrimidines and non-essential amino acids as well as NADPH required for fatty acid synthesis and maintenance of the cell’s redox state. Although the pathway is typically up-regulated in cancer cells in order to support sustained energy production and biosynthesis, our results suggest that NOX1 acts to inhibit anaplerosis and redirect glutamate from the TCA cycle towards conversion to aspartate (Fig. 6). In line with our findings, a non-canonical K-Ras-dependent pathway of glutamine metabolism has been recently described in human pancreatic ductal adenocarcinoma cells, where glutamine is converted to glutamate in the mitochondria, which instead of fueling the TCA cycle is converted to aspartate [49]. The aspartate is transported to the cytoplasm and converted to OAA then to malate and finally to pyruvate while also generating NADPH [49]. In support, our analysis showed that NOX1 levels were positively correlated to cytosolic aspartate aminotransferase (cAspAT, EC = 2.6.1.1) which converts cytosolic aspartate into OAA supporting a non-canonical metabolic transformation of glutamine similar to the one described in pancreatic cells (Fig. 6). Our results suggest that HepG2 cells undergo a reprogramming of glutamine metabolism and NOX1 plays a role in this process by regulating the abundance of two of the key enzymes.

### Nucleotide synthesis

We further found that NOX1 levels were correlated with the abundance of GMP reductase 2 (GMPR2, EC = 1.7.1.7.), which catalyzes the NADPH-dependent irreversible deamination of GMP to IMP. By reconverting GMP and AMP to inosine monophosphate (IMP) which is a precursor of both nucleotides, cells can maintain the intracellular balance of adenine and guanine nucleotides and our result suggests that NOX1 is involved in regulating this balance (Fig. 6). Another way in which NOX1 may support nucleotide synthesis is by increasing the availability of precursors for the pentose phosphate pathway through the inhibition of glycogen synthesis and the conversion of lactate and TCA cycle intermediates into PEP (see above). Both of these two mechanisms mediated by NOX1 are synergic with the AMP and GMP feedback inhibition mechanisms which regulate the biosynthesis of purine nucleotides. Thus, since ATP is required for the conversion of IMP to GMP, the lower concentration of ATP caused in tumor cells by the reduced utilization of glucose in the TCA cycle, will also promote AMP over GMP synthesis. Collectively, all these mechanisms will operate to restore ATP levels and reestablish the cellular balance between ATP and GTP, which is frequently disturbed in tumor cells.

### Lipid metabolism

Tumor cells frequently have dysregulated lipid metabolism due to enhanced fatty acid and lipid biosynthesis on the one hand and augmented β-oxidation of fatty acids for energy production on the other hand [50]. Our study shows that reduced NOX1 levels result in diminished levels of cytosolic hydroxymethylglutaryl-CoA synthase (HMG-CoA synthase, EC = 2.3.3.10). Thus, NOX1 may contribute to remodeling of lipid metabolism in hepatic tumor cells since HMG-CoA synthase catalyzes the second step of the mevalonate biosynthesis, leading to lipid, steroid (including cholesterol) and isoprenoid biosynthesis. Increased levels of mevalonate have been observed in several tumor cell lines to be associated with increased cellular proliferation and tumor growth [51], and altered levels of HMG-CoA synthase have been described in in HCC [52]. Liver tumor cells have also been shown to contain higher levels of cholesterol as compared to normal liver tissue [52,53]. Moreover, cholesterol rich rafts are critical for receptor triggered intracellular signaling, and cholesterol depletion results in Akt inactivation and apoptosis [51].

We also found that NOX1 protein levels were inversely correlated with the levels of long chain fatty acid-CoA ligase 1 (Acsl1, EC = 6.2.1.3). This enzyme facilitates the uptake and catalyzes the activation of C10-C22 fatty acids which are needed for lipid biosynthesis, but also for lipid degradation and energy production via beta-oxidation [54]. There is mixed evidence regarding the destination of the Acsl1-activated fatty acids in liver cells. Although the loss of Acsl1 from mouse liver impairs both triglyceride synthesis and beta-oxidation [55], overexpression of the enzyme in HepG2 cells and rat liver enhances triglyceride synthesis without altering fatty acid oxidation [56]. Interestingly, in humans its expression is lower in HCC as compared to normal liver [57] suggesting a role in the tumor-specific remodeling of lipid metabolism.

### NOX1 and protein synthesis and turnover

Tumor cells have increased rates of protein synthesis and turnover and our results suggest that NOX1 might play a role in controlling these processes in hepatic tumor cells. Among the proteins whose abundances were correlated with the abundance of NOX1 was the elongation factor 2 (EF2). This protein catalyzes the GTP-dependent ribosomal translocation of the nascent protein chain during translation. Its expression is tightly regulated during the cell cycle and has been reported to increase in different cancers [58]. Interestingly, EF2 is inactivated upon phosphorylation by EF2 kinase, a process promoted by oxidative stress, whereby protein synthesis is completely halted [59]. However, our 2DE data showed that EF2 in NOX1 levels were positively correlated, suggesting that NOX1 supports protein synthesis and might play a role in the regulation of cell cycle progression.

The reduction of NOX1 levels also reduced the expression of Mep50(p44), a WD-repeat containing steroid receptor co-activator involved in the regulation of androgen receptor (AR) and estrogen receptor (ER) target genes [60] and a component of the 20S protein arginine methyltransferase complex (methylosome), which is involved in the assembly of the spliceosome [61]. Mep50 function is regulated by its subcellular localization, which exhibits cell type specific changes during development and tumorigenesis [60,62], but the specific role of Mep50 in liver and hepatoblastoma remains unclear.

NOX1 expression was also correlated with the expression of the SET protein, a member of the INHAT (inhibitor of histone acetyltransferase) complex that binds un- or hypo-acetylated histones preventing their acetylation. Recently, the protein has been shown to inhibit p53 acetylation and thus to induce cellular proliferation and to block p53-mediated cell cycle arrest and apoptosis in response to cellular stress [63]. SET protein is a member of the SET complex, involved in the regulation of gene expression, DNA replication and repair, and cellular response to oxidative stress. The complex is ubiquitously expressed and up-regulated in ovarian cancer [64] and HCC [65]. In HCC, accumulation of SET protein has been correlated with low survival [66]. Further investigations are required in order to explain the roles of the SET protein in HepG2 cells and its regulation by NOX1.

Finally, the abundance of the PA28β proteasome activator was correlated with that of NOX1. This protein is part of the 11S regulatory particle of the immunoproteasome which plays an important role in antitumor immunity due to its enhanced antigen generating capacity [67]. The immunoproteasome is also involved in the efficient degradation of oxidatively damaged proteins and thus in the maintenance of protein homeostasis and the preservation of cell viability under cytokine induced oxidative stress conditions [68].

### Other protein functions

Two members of the annexin family (A2 and A3), both known to be over-expressed in many cancers including HCC [69–71], were reduced in NOX1 depleted HepG2 cells. Annexin A2 has various functions, including exocytosis, endocytosis and membrane trafficking, cell division and proliferation [72]. It has previously been linked to cholesterol metabolism [73] and its association to the membrane is cholesterol dependent [74], which is in line with our supposition that NOX1 may support cholesterol synthesis (see the chapter on lipid metabolism). Annexin A2 overexpression and phosphorylation has been related to malignant transformation, progression and differentiation in HCC [75]. Conversely, reduced annexin A2 expression decreases proliferation and induces apoptosis [76], possibly through a p53 related mechanism [77]. Extracellular annexin A2 has been shown to mediate the degradation of the extracellular matrix and to promote angiogenesis and tumor growth [72]. Since annexin A2 can be up-regulated by H_2_O_2_ [76] our study suggests that NOX1 might induce annexin A2 via a ROS-dependent mechanism and thus control cell proliferation.

Annexin A3 is necessary for DNA synthesis in cultured hepatocytes [78] and is part of the signaling cascade involved in liver regeneration [79]. The protein has been associated with tumor progression in lung adenocarcinoma [80]. Importantly, similarly to annexin A2, annexin A3, has been shown to act as an angiogenic mediator by inducing VEGF production through the HIF-1 pathway [81]. Since ROS derived from NADPH oxidases have been shown to increase VEGF, the HIF-1 pathway and angiogenesis [82], induction of annexin A2 and/or annexin A3 by NOX1 might contribute to these responses.

NOX1 levels were further correlated with the levels of albumin, the most abundant protein in the adult serum. Albumin plays a role in maintaining the redox balance and can scavenge ROS and RNS thus leading to inhibition of apoptosis and activation of the Akt pathway [83,84]. Both HCCs and HBs (including HepG2 cells) produce less albumin than normal human liver [85,86]. In cancer patients (including HCC) low serum albumin predicts a poor response to treatment and poor survival [87] and a large proportion of HCC patients have ROS damaged serum albumin [88].

NOX1 levels were also correlated with the levels of α-fetoprotein (AFP), a major serum protein produced by the fetal liver which is repressed upon hepatocyte maturation but reactivated in liver malignancies [89]. In HCC, AFP serum levels correlate directly with disease progression and aggressiveness [90] and inversely with the degree of cell differentiation [90,91]. In HB on the other hand, low AFP levels are associated with poor response to chemotherapy and poor prognosis [90]. The decrease of AFP levels in NOX1 depleted HepG2 cells, known to be AFP positive [92], suggests an involvement of NOX1 in cell differentiation [93]. Although there is a large variation in AFP and albumin production among HCCs and HBs [94], the expression levels of the two proteins are inversely correlated in hepatocytes during development, and malignant transformation typically reverses their normal ratio by increasing AFP levels and decreasing albumin levels. However, NOX1 apparently supports the expression of both proteins in HepG2 cells, paralleling previous observations in HCC [95].

NOX1 reduced cells expressed increased levels of fascin, an actin bundling protein with an important role in cell adhesion, motility and migration [96]. Fascin is weakly expressed in normal liver [97] but over-expressed during malignant transformation and progression in several cancers, including HCC [98,99] and HB [100]. The protein has been associated with tumor aggressiveness [98] and is a significant indicator of poor prognosis in HCC [99]. The protein can increase the invasiveness of several HCC cell lines, although not of the HCC line Hep3B or of the HB line HepG2 [100]. In colon adenocarcinoma cells NOX1 was shown to act as a switch between random and directional migration and to control the directionality of cell migration [101,102]. Interestingly, this mechanism involved α3β1 integrin, whose interaction with the extracellular matrix proteins induces the formation of fascin micro spikes and cell scattering [103].

Our investigations also showed that NOX1 levels are positively correlated with the levels of Hep27 (EC = 1.1.1.184), a NADPH-dependent dicarbonyl reductase initially isolated from growth-arrested HepG2 cells [104]. Hep27 functions as a reactive alpha-carbonyl scavenging enzyme [105] with a protective role against oxidative stress [106]. The mature mitochondrial Hep27 can be partially translocated to the nucleus where it binds Mdm2 promoting p53 stabilization [107].

## Conclusion

Cells undergoing malignant transformation experience extensive metabolic remodeling (known as Warburg effect) designed to support the characteristically high proliferation rates [38]. The proteomic profiling of NOX1 depleted HepG2 cells suggested that NOX1 mediates the remodeling of the cellular metabolism such as to allow available resources to be diverted towards biosynthesis of building blocks required for cell growth. Thus, our results show that NOX1 is involved in the regulation of glucose and glutamine utilization as well as lipid, protein and nucleotide synthesis. Further protein functions dysregulated upon the depletion of NOX1 also support previous reports on the role of this enzyme in regulating tumor growth.

## Supporting Information

S1 Table Proteins dysregulated in NOX1 depleted HepG2 cells. Details are provided on the MS identification of the differentially expressed proteins including accession numbers, predicted and experimental MW and pI, sequence coverage, peptide sequences and MASCOT scores.
(XLSX)

S1 Fig Functional classification of proteins. Proteins dysregulated in shNOX1 HepG2 cells have been classified according to their cellular function.
(PDF)

S2 Fig Protein localisation. Proteins dysregulated in shNOX1 HepG2 cells have been classified according to their cellular localisation.
(PDF)

S3 Fig Western blot analysis of NOX1 depleted Huh7 cells. NOX1 depleted Huh7 cells express lower levels of UDPGP and GDH1 as compared to control cells. Protein abundance was determined by Western blot analysis in Huh7 cells expressing shNOX1 plasmid or a control shRNA.
(PDF)

S4 Fig Metabolic activity of NOX1 depleted HepG2 cells. NOX1 depleted HepG2 cells display lower metabolic rates as compared to control cells. AlamarBlue fluorescence assay was performed over a time course of 6 days. The difference in slopes between NOX1 depleted cells and control cells was tested using a mixed effect model with replicate (N = 3) as random factor.
(PDF)

S1 File Full pictures of 2DE gels and Western blots. Full pictures are provided for all 2DE gels and Western blots analyzed and presented.
(PDF)
