# Supplementary material for: NOX1 Supports the Metabolic Remodeling of HepG2 Cells
Source: PLoS One. 2015 Mar 25;10(3):e0122002. doi: 10.1371/journal.pone.0122002 (PMC4373763; doi:10.1371/journal.pone.0122002)
Supplement: S1 Fig — Proteins dysregulated in shNOX1 HepG2 cells have been classified according to their cellular function. (PDF) [file pone.0122002.s002.pdf]

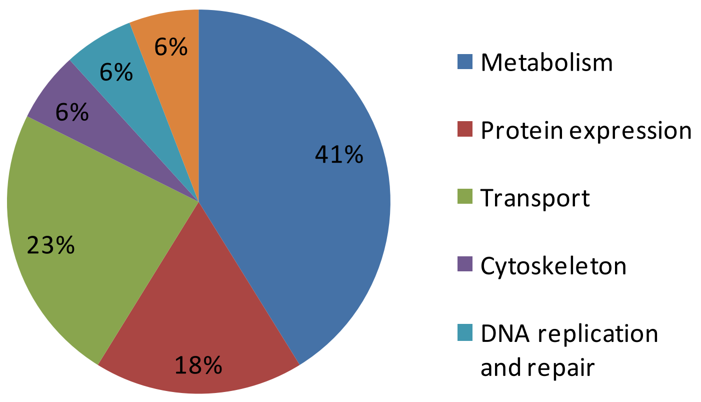

S1 Figure. Functional classification of proteins. Proteins dysregulated in shNOX1 HepG2 cells have been classified according to their cellular function.
