# Supplementary material for: NOX1 Supports the Metabolic Remodeling of HepG2 Cells
Source: PLoS One. 2015 Mar 25;10(3):e0122002. doi: 10.1371/journal.pone.0122002 (PMC4373763; doi:10.1371/journal.pone.0122002)
Supplement: S3 Fig — NOX1 depleted Huh7 cells express lower levels of UDPGP and GDH1 as compared to control cells. Protein abundance was determined by Western blot analysis in Huh7 cells expressing shNOX1 plasmid or a control shRNA. (PDF) [file pone.0122002.s004.pdf]

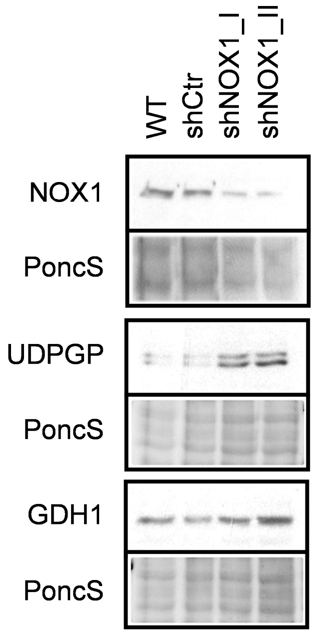

S3 Figure. Western blot analysis of NOX1 depleted Huh7 cells. NOX1 depleted Huh7 cells express lower levels of UDPGP and GDH1 as compared to control cells. Protein abundance was determined by Western blot analysis in Huh7 cells expressing shNOX1 plasmid or a control shRNA.
