# Supplementary material for: NOX1 Supports the Metabolic Remodeling of HepG2 Cells
Source: PLoS One. 2015 Mar 25;10(3):e0122002. doi: 10.1371/journal.pone.0122002 (PMC4373763; doi:10.1371/journal.pone.0122002)
Supplement: S4 Fig — NOX1 depleted HepG2 cells display lower metabolic rates as compared to control cells. AlamarBlue fluorescence assay was performed over a time course of 6 days. The difference in slopes between NOX1 depleted cells and control cells was tested using a mixed effect model with replicate (N = 3) as random factor. (PDF) [file pone.0122002.s005.pdf]

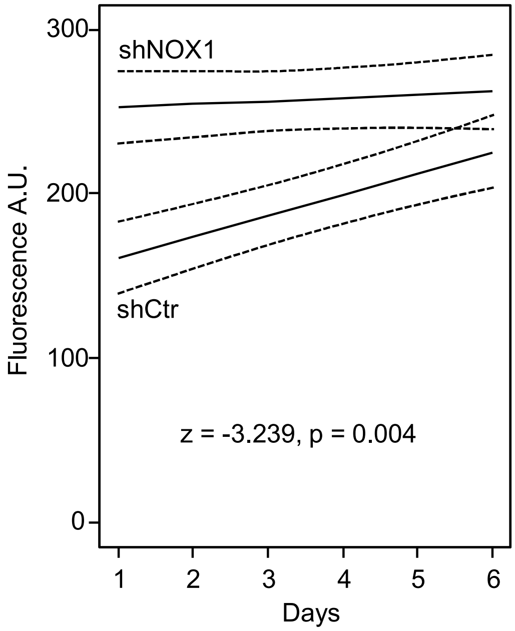

S4 Figure. Metabolic activity of NOX1 depleted HepG2 cells. NOX1 depleted HepG2 cells display lower metabolic rates as compared to control cells. AlamarBlue fluorescence assay was performed over a time course of 6 days. The difference in slopes between NOX1 depleted cells and control cells was tested using a mixed effect model with replicate (N = 3) as random factor.
