# Supplementary material for: NOX1 Supports the Metabolic Remodeling of HepG2 Cells
Source: PLoS One. 2015 Mar 25;10(3):e0122002. doi: 10.1371/journal.pone.0122002 (PMC4373763; doi:10.1371/journal.pone.0122002)

shCtr 2DE gels IPG 4-7

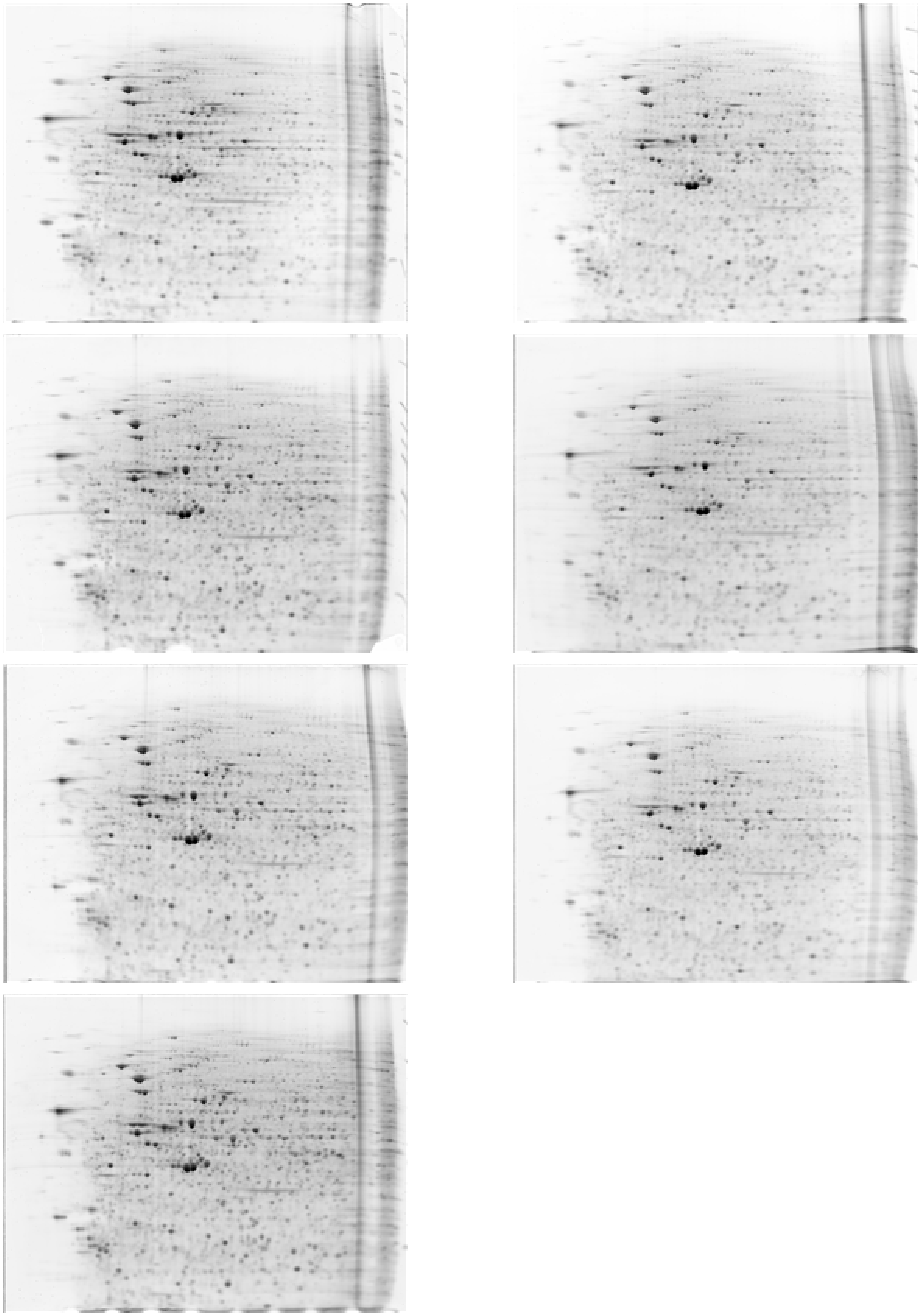

shNOX1 2DE gels IPG 4-7

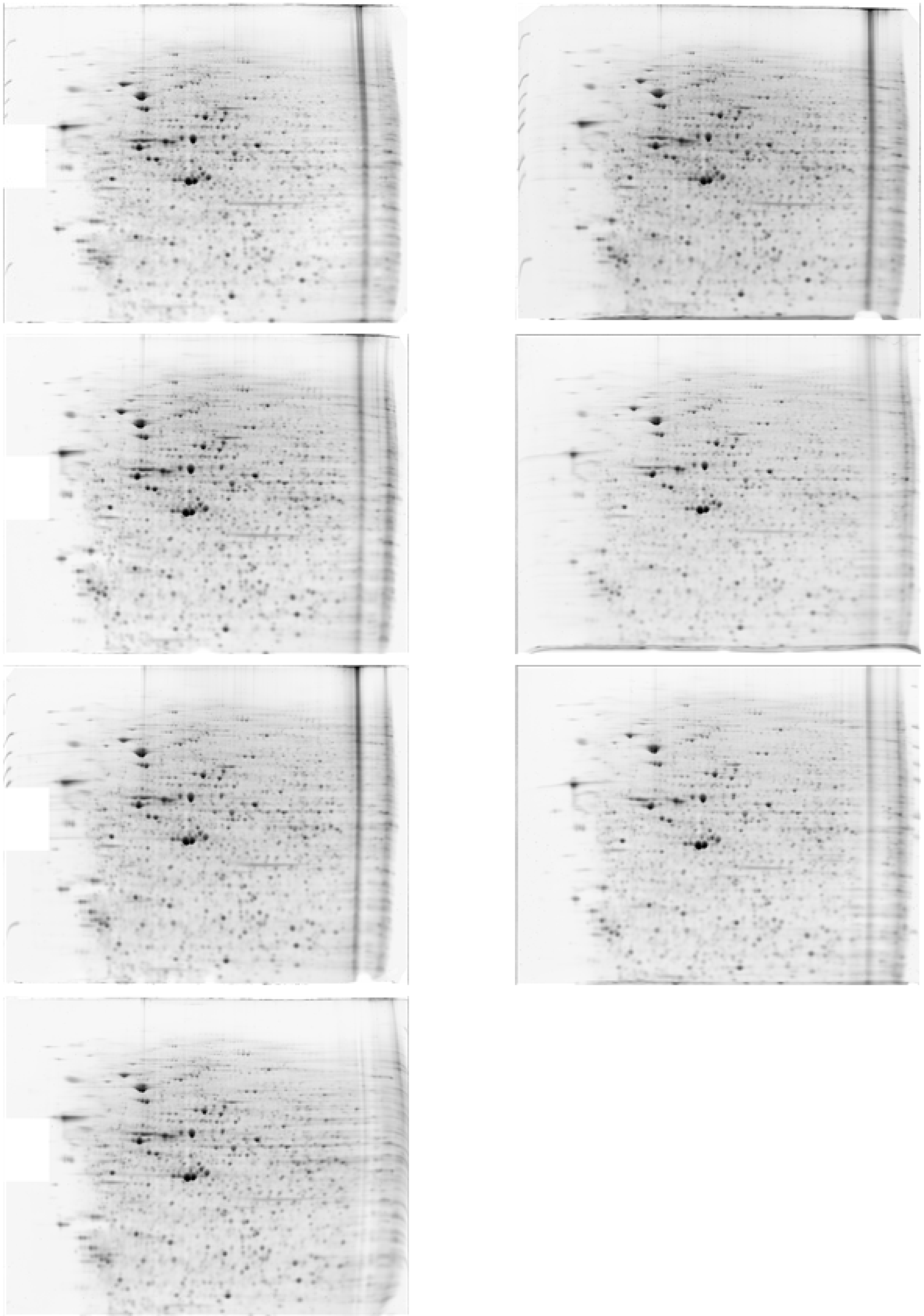

shNOX1 2DE gels IPG 4-7
Independent line used for validation

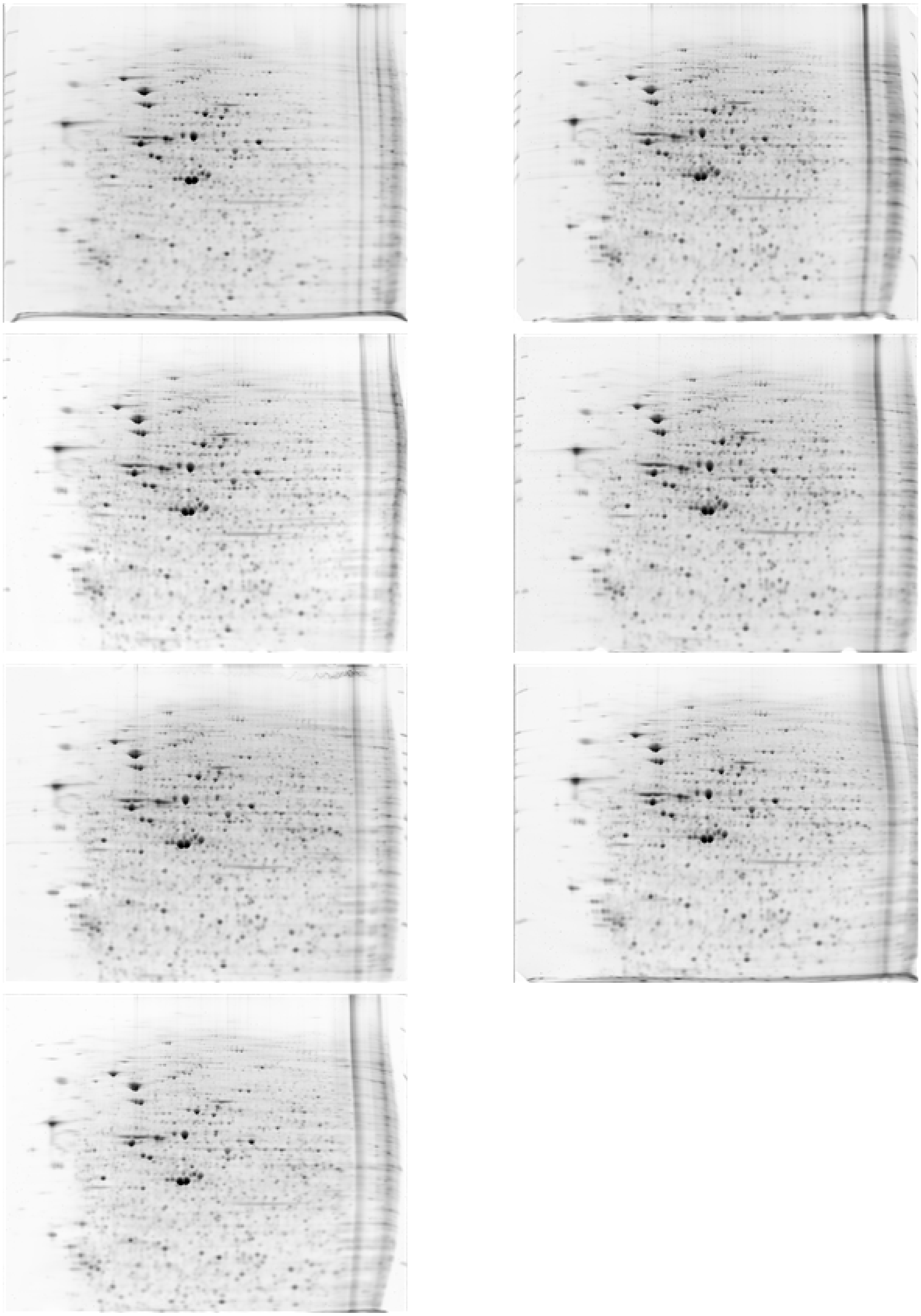

Wild type HepG2 2DE gels IPG 4-7
used for validation

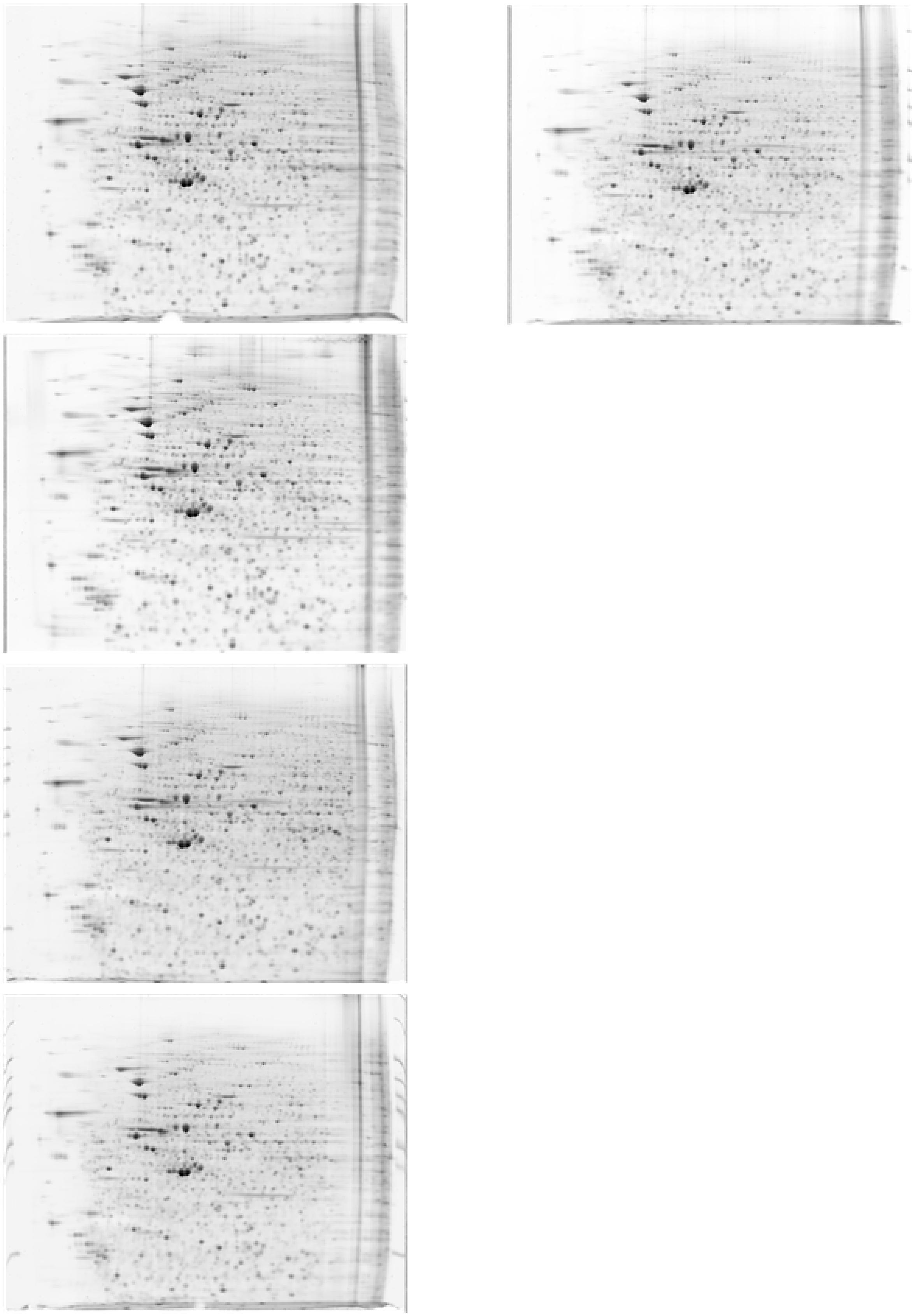

shCtr 2DE gels IPG 6-11

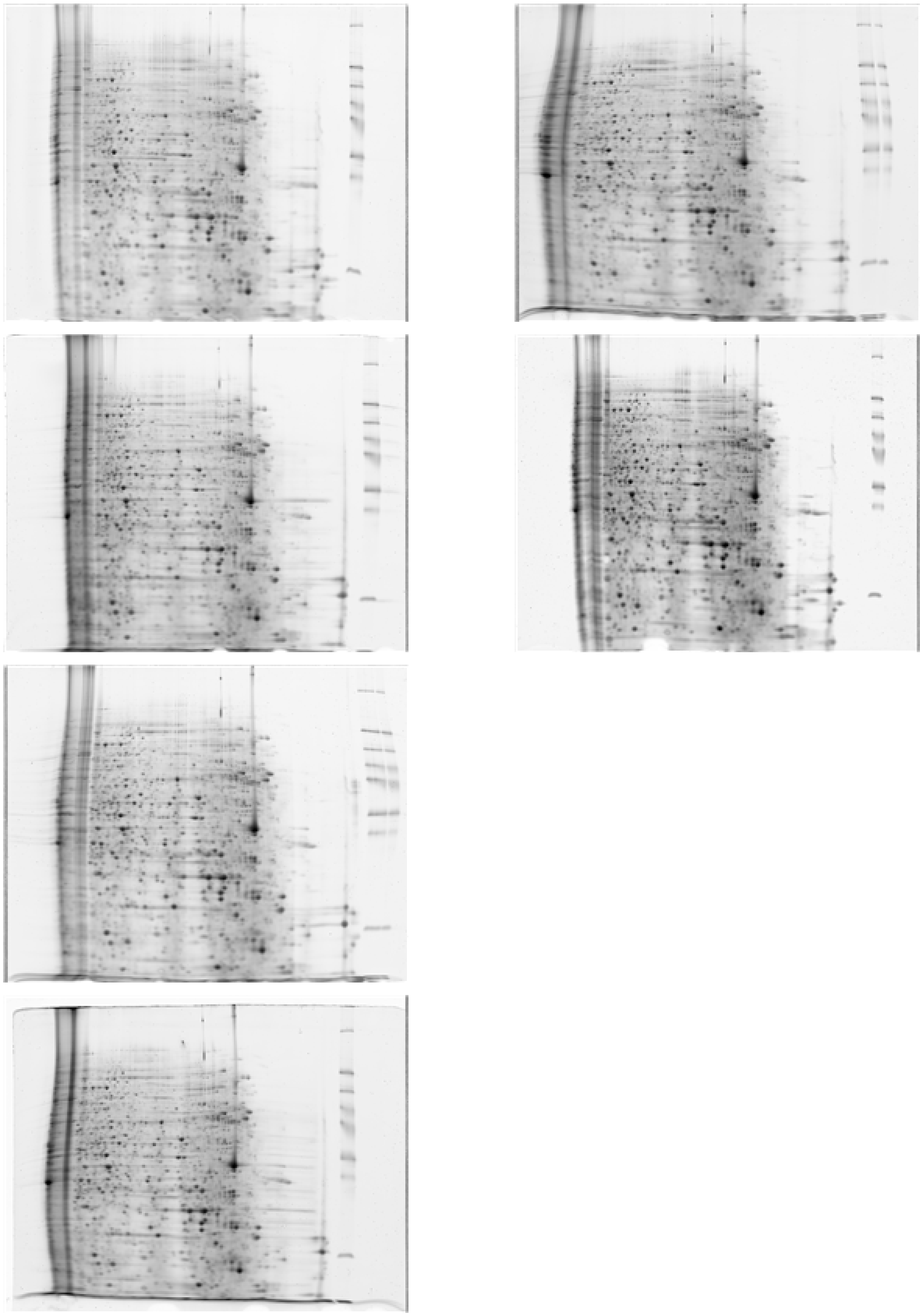

shNOX1 2DE gels IPG 6-11

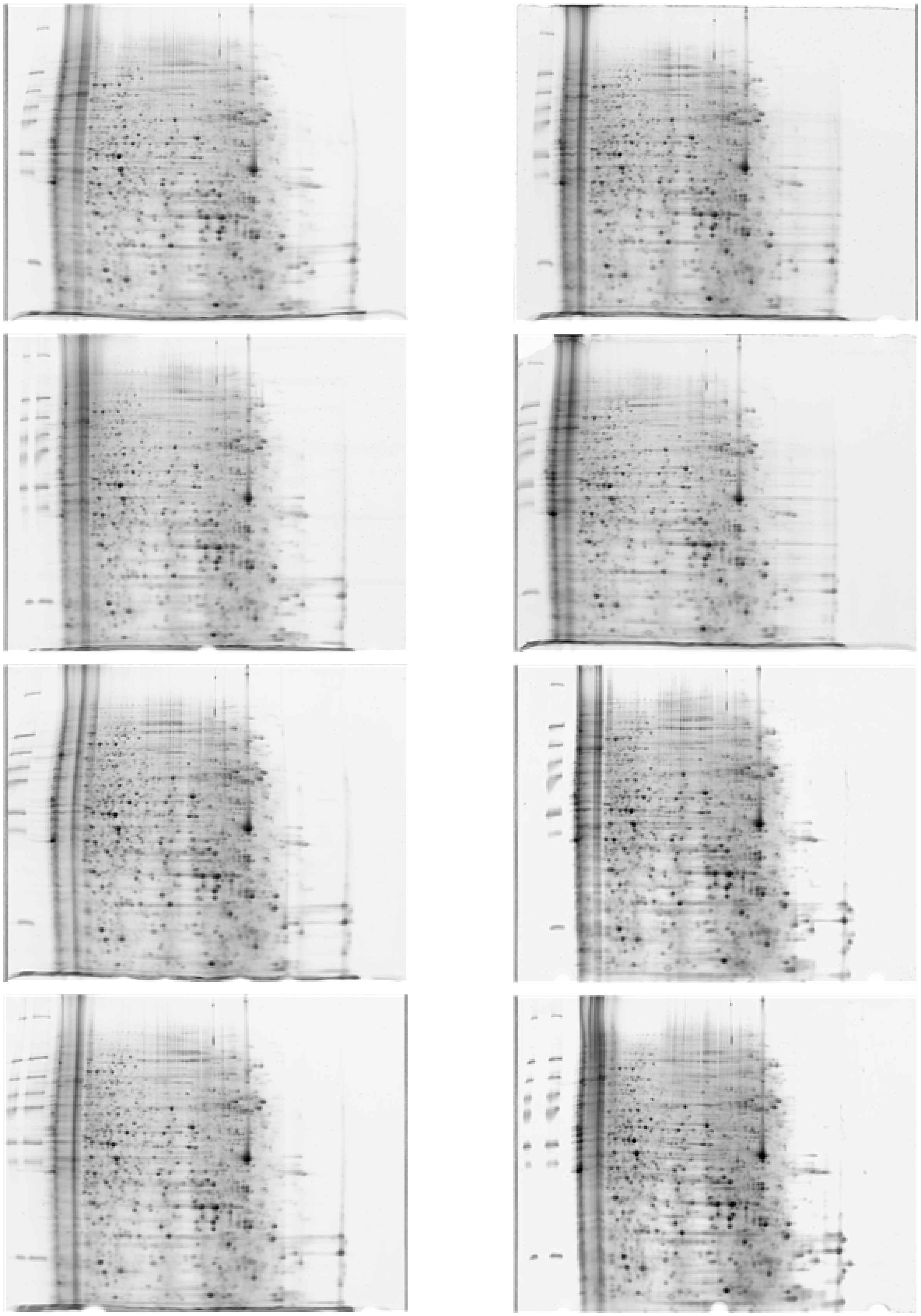

shNOX1 2DE gels IPG 6-11
Independent line used for validation

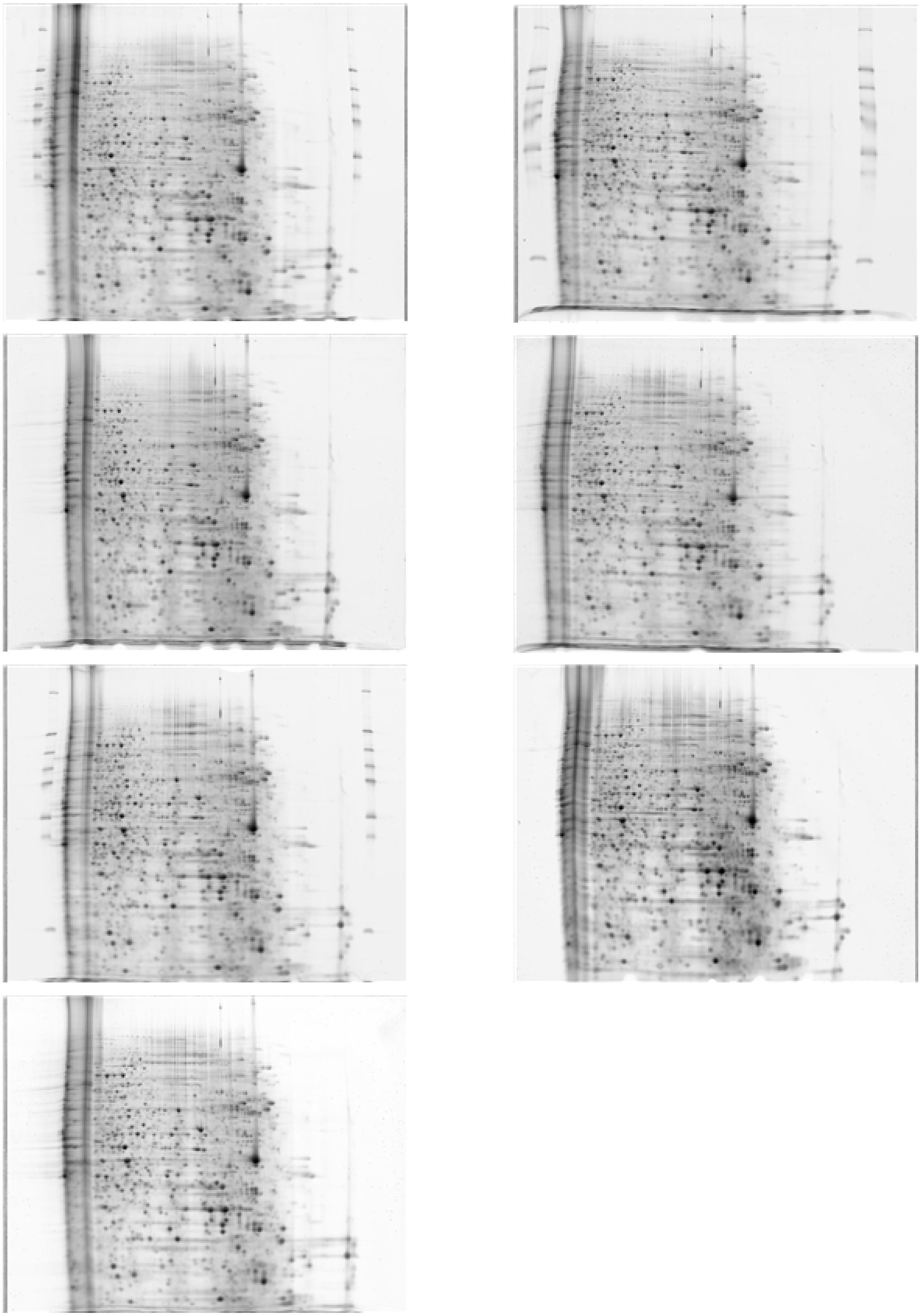

Wild type HepG2 2DE gels IPG 6-11 used for validation

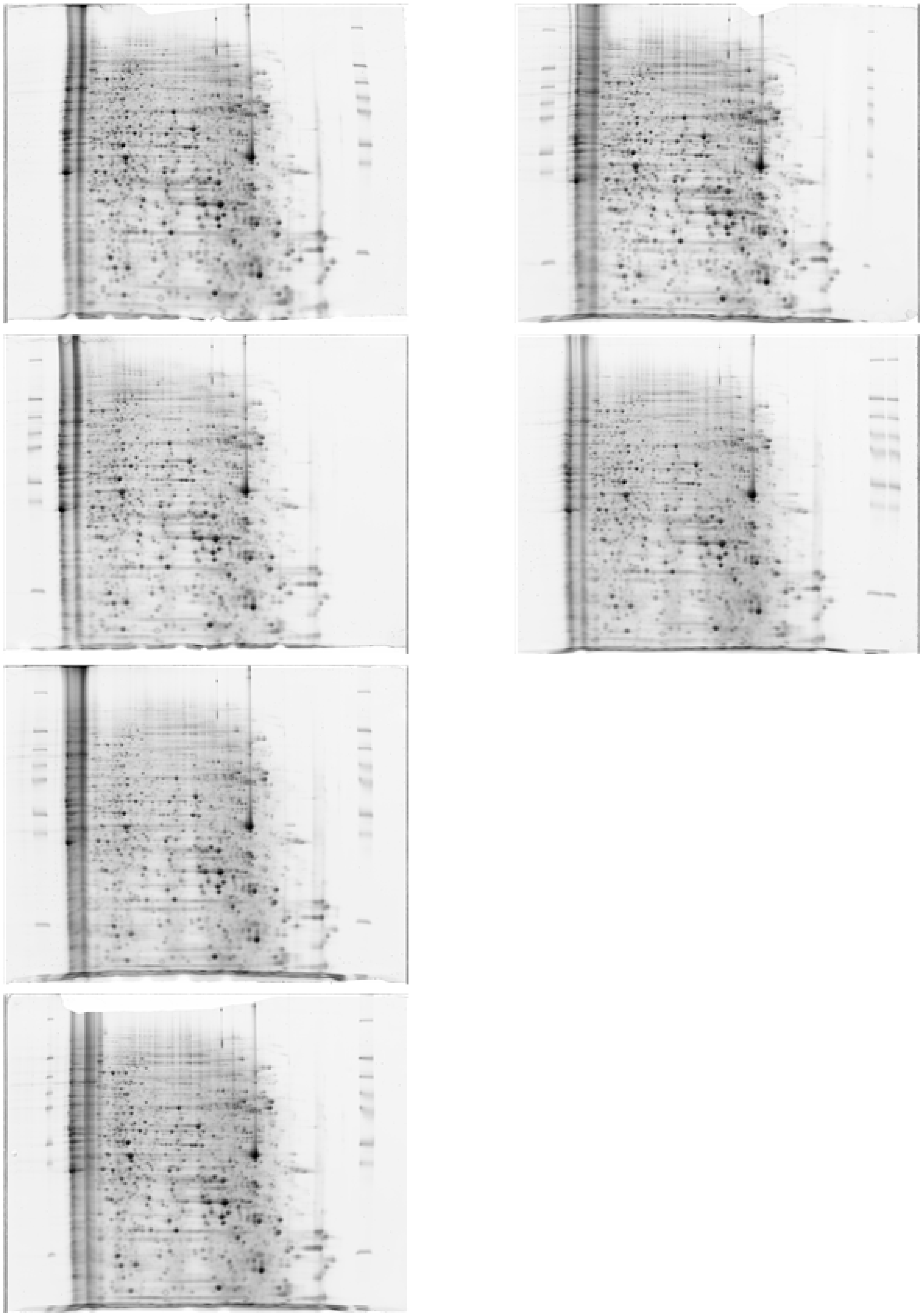

Western Blot validation of differentially expressed proteins

Left: WB, Right: PonceauS

SET protein

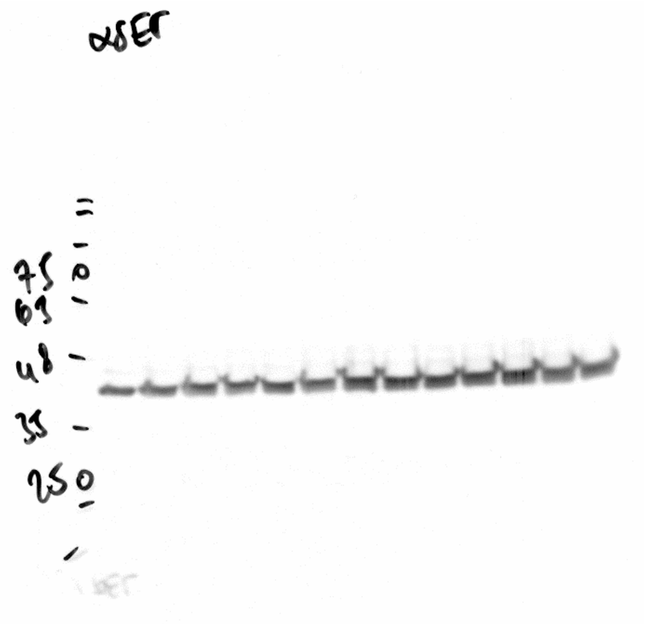

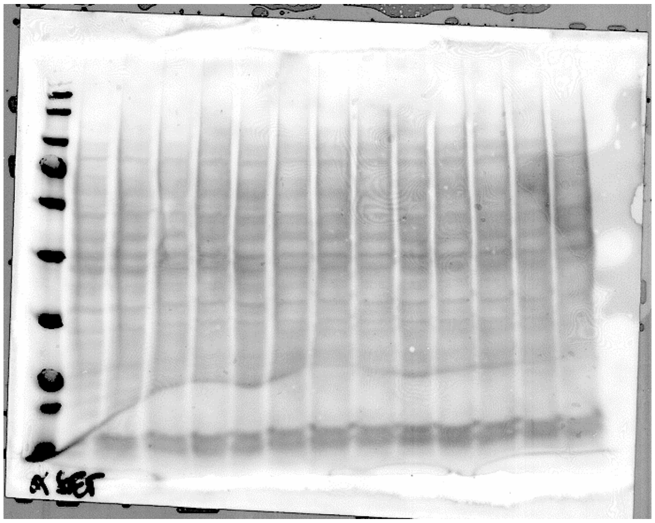

HMGC

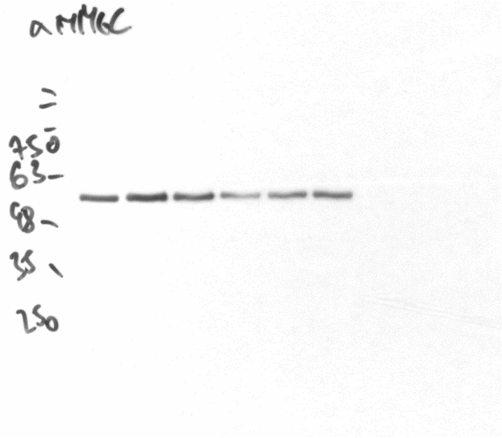

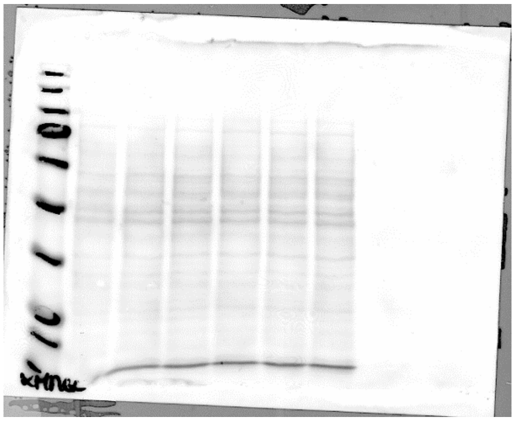

AFP

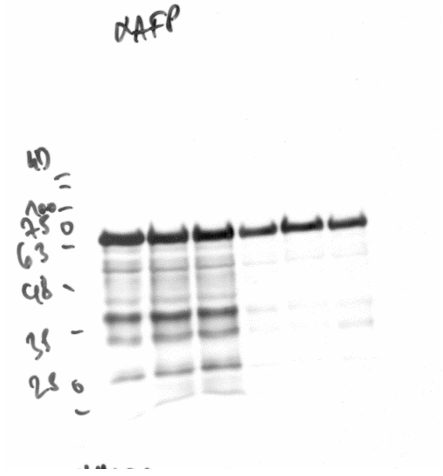

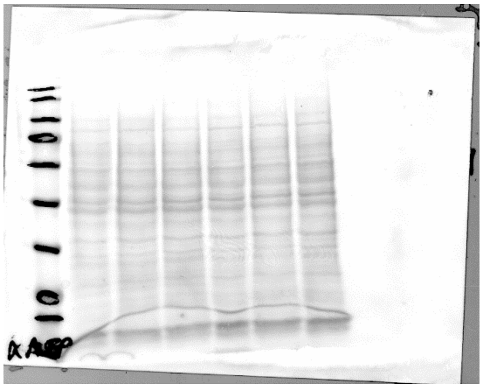

Western Blot validation of differentially expressed proteins

Left: WB, Right: PonceauS

Acsl1

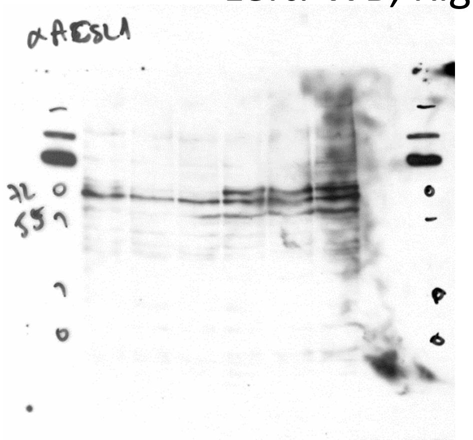

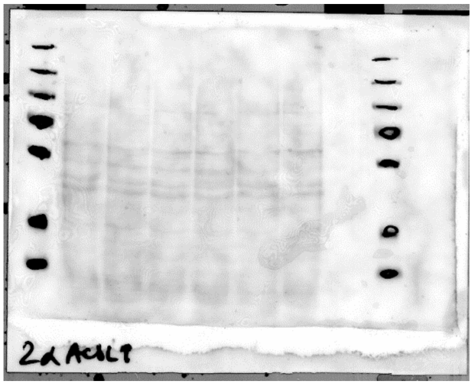

Fascin

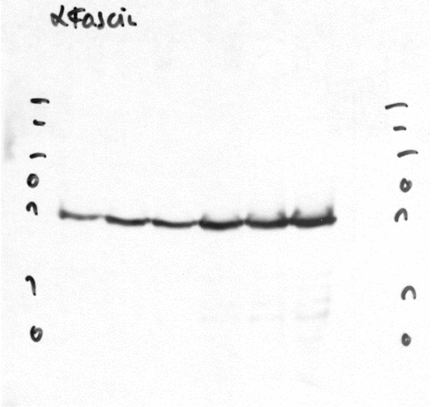

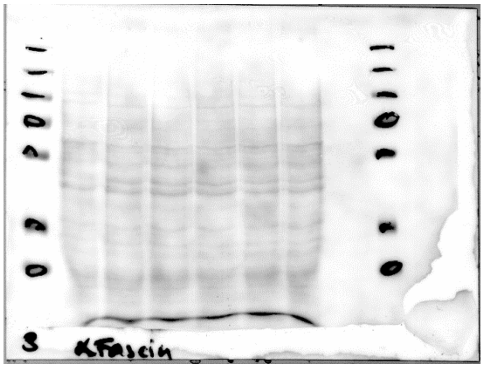

GDH1

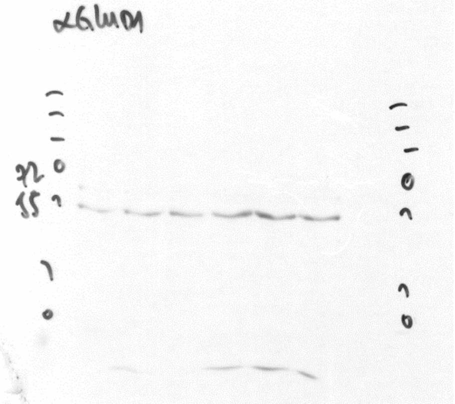

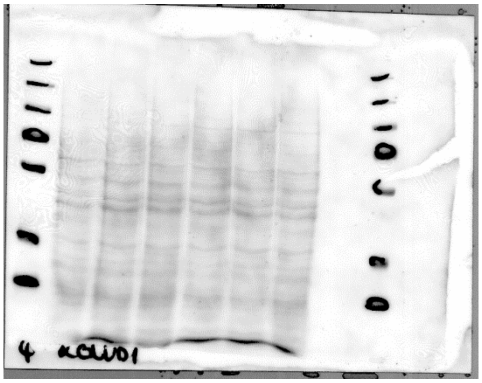

UDPGP

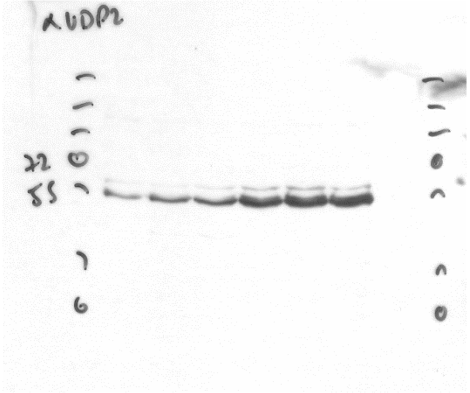

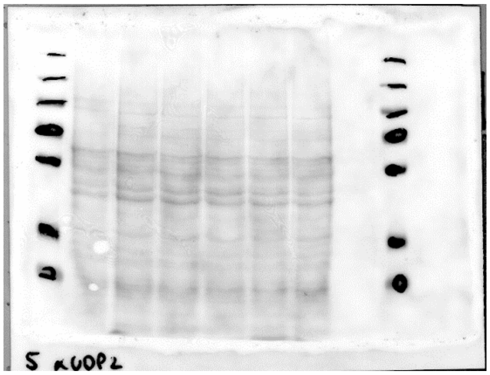

Western Blot

Left: WB, Right: PonceauS

αNOX1
(Fig. 1)

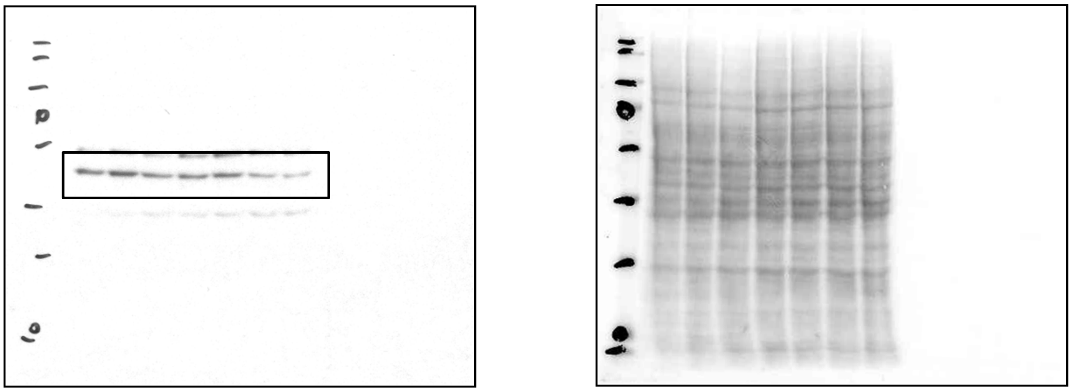

αNOX1
(Fig. 5)

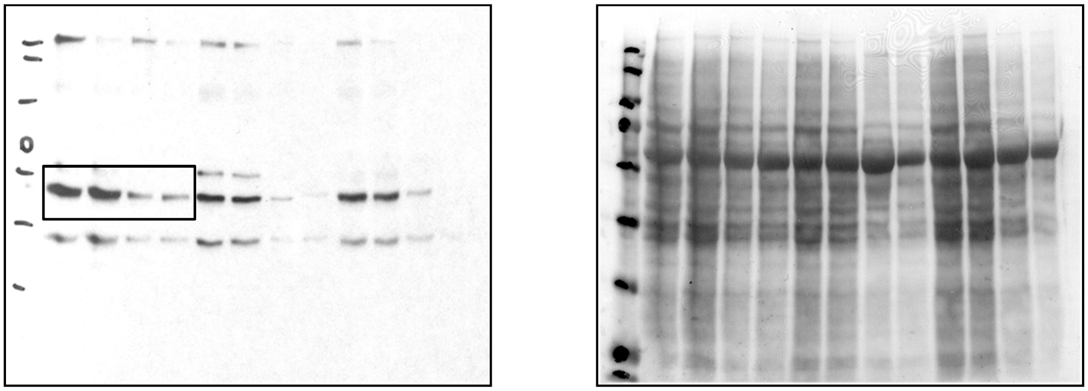

Supplement: S1 File — Full pictures are provided for all 2DE gels and Western blots analyzed and presented. (PDF) [file pone.0122002.s006.pdf]
